# Interactive Effects of Melatonin and Nitrogen Improve Drought Tolerance of Maize Seedlings by Regulating Growth and Physiochemical Attributes

**DOI:** 10.3390/antiox11020359

**Published:** 2022-02-11

**Authors:** Shakeel Ahmad, Guo-Yun Wang, Ihsan Muhammad, Yu-Xin Chi, Muhammad Zeeshan, Jamal Nasar, Xun-Bo Zhou

**Affiliations:** Guangxi Colleges and Universities Key Laboratory of Crop Cultivation and Tillage, National Demonstration Center for Experimental Plant Science Education, Agricultural College of Guangxi University, Nanning 530004, China; shakeel@gxu.edu.cn (S.A.); 2017401007@st.gxu.edu.cn (G.-Y.W.); ihsan@stumail.nwu.edu.cn (I.M.); byndcx123@163.com (Y.-X.C.); p2020011@gxu.edu.cn (M.Z.); jnasar@gxu.edu.cn (J.N.)

**Keywords:** melatonin, nitrogen, drought stress, enzyme activity, maize

## Abstract

Melatonin plays an important role in numerous vital life processes of animals and has recently captured the interests of plant biologists because of its potent role in plants. As well as its possible contribution to photoperiodic processes, melatonin is believed to act as a growth regulator and/or as a direct free radical scavenger/indirect antioxidant. However, identifying a precise concentration of melatonin with an optimum nitrogen level for a particular application method to improve plant growth requires identification and clarification. This work establishes inimitable findings by optimizing the application of melatonin with an optimum level of nitrogen, alleviating the detrimental effects of drought stress in maize seedlings. Maize seedlings were subjected to drought stress of 40–45% field capacity (FC) at the five-leaf stage, followed by a soil drenching of melatonin 100 µM and three nitrogen levels (200, 250, and 300 kg ha^−1^) to consider the changes in maize seedling growth. Our results showed that drought stress significantly inhibited the physiological and biochemical parameters of maize seedlings. However, the application of melatonin with nitrogen remarkably improved the plant growth attributes, chlorophyll pigments, fluorescence, and gas exchange parameters. Moreover, melatonin and nitrogen application profoundly reduced the reactive oxygen species (ROS) accumulation by increasing maize antioxidant and nitrogen metabolism enzyme activities under drought-stress conditions. It was concluded that the mitigating potential of 100 µM melatonin with an optimum level of nitrogen (250 kg N ha^−1^) improves the plant growth, photosynthetic efficiency, and enzymatic activity of maize seedling under drought-stress conditions.

## 1. Introduction

The world is currently confronting several environmental concerns, including rising temperatures, rising CO_2_ levels, water scarcity, and soil degradation [1]. Water shortage is the most important environmental limitation worldwide that seriously affects agriculture production [2]. Water scarcity has a variety of consequences on plants, including reduced membrane integrity, osmotic effects, pigment content, plant development, photosynthetic activity, and reducing production [3,4]. Drought stress has an effect on plant growth and development as well as the ecosystem [5]. Plant water interactions and water efficiency are negatively impacted by drought stress. Depending on the degree and duration of the drought stress, and the plant’s growth period, drought has diverse effects on plants [6]. Plants’ drought stress response mechanisms are extremely complicated, and they differ greatly depending on plant species, growth stage, and duration of water scarcity [7]. According to statistics, in the last few years, drought stress seriously affected agricultural food production by 15.6 to 48.5% [8]. Consequently, one of the main focuses of modern plant research is to improve drought tolerance in crop species. Maize is an important food, fodder, and industrial raw material in China; hence, it impacts agriculture and the economy [9]. Maize is more sensitive to drought stress and requires more water than other crop species. Therefore, water scarcity is the major factor globally in reducing maize production [10]. As a result of global warming, water scarcity has worsened in recent years, posing a serious threat to food production and agriculture sustainability, as well as global food security [11].

Nitrogen (N) is the major source and a vital nutrient for plant growth and development because it affects the composition of proteins, nucleic acids, hormones, and other critical compounds [12,13]. To meet the agricultural demands of a growing population, synthetic N fertilizer application has increased dramatically in the last 50 years [14]. Nitrogen fertilizer application rates have recently increased considerably in China’s intensive agricultural systems, mostly for crop production [13]. Whereas the higher N fertilizer input has increased crop productivity, reduced plant N utilization efficiency and excess N can have serious consequences for the environment and human health [15,16]. Reducing the nitrogen fertilizer ratio by maintaining crop growth and production is an important challenge for agriculture’s long-term survival [17].

Melatonin (*N*-acetyl-5-methoxytryptamine, MT), a low-molecular-weight molecule containing an indole ring in its structure, has been detected in organisms that are evolutionarily distant [18]. This conserved molecule can be found in all living organisms, from bacteria to animals, and plants utilize it for various functions [15,18]. In plants’ systems, the most important function of melatonin is to protect them from biotic and abiotic stresses such as cold, drought, polyethylene glycol -induced osmotic stress, and heat [19,20]. Melatonin can also be found in both animals and plants and is helpful to human health [21]. As a result, using melatonin as a plant growth regulator in crop production would pose no dangers to the environment or human health. In recent years, these unique properties have attracted individuals to employ it as a natural bio stimulator in crop development [20,22,23]. Melatonin has recently been discovered to improve plant nutrient uptake and protect plants from a variety of biotic and abiotic stressors [13]. Melatonin has been proven to improve the root/shoot ratio and regulate lateral root development, and a strong root system increases the nutrient uptake under drought-stress conditions [24]. Drought and salt stress make it harder for plants to absorb nutrients, and melatonin helps to reduce these problems by improving nutrient absorption and metabolism [25]. Nitrogen metabolism, being one of the most critical nutrients for plant growth, has been shown in some plant species to be regulated by the application of melatonin [15,18]. Exogenous melatonin improves cucumber seedling tolerance to high nitrate stress by increasing the activity of enzymes involved in nitrogen metabolism [15]. In melon seedlings which suffered sub-low temperatures, exogenous MT significantly decreased the leaf NH_4_^+^-N content by improving the activities of N metabolism-related enzymes, such as glutamine synthetase (GS) and nitrate reductase (NR) activities [26]. Melatonin could maintain nitro-oxidative homeostasis by regulating N species at the enzymatic and/or transcript level in drought-stressed alfalfa [27]. Melatonin could also protect organisms from oxidative stress by interacting with reactive oxygen and nitrogen species, resulting in a highly effective free radical scavenging cascade [13]. Melatonin’s effects on plant N uptake and metabolism, particularly in crop plants, are a little unexplored. Furthermore, research on MT as a plant growth regulator that can be applied to crop production, MT performance, and underlying processes on crop development is ongoing.

Maize is an important food and fodder crop that impacts global food security. Exogenous application of MT enhanced maize drought tolerance by relieving photosynthetic inhibition and oxidative damage caused by abiotic stresses [7,19]. Melatonin has been discovered to increase photosynthetic carbon assimilation and antioxidant enzyme activity in maize under abiotic stress conditions [28]. Maize growth and productivity are hampered by a lack of nitrogen [29]. However, to date, few studies have explored the performance and function of melatonin combined with nitrogen-based on evaluation and N absorption of metabolism in cereal crops under drought-stress conditions [11]. Therefore our objective was to examine the effect of melatonin treatment as a root drenching with different N levels on the growth and the performance of maize seedlings subjected to drought-stress conditions. Thus, in this study, a comprehensive understanding of the role of exogenous MT treatments with different levels of N was provided to improve the maize seedlings’ growth in drought-stress conditions.

## 2. Materials and Methods

### 2.1. Plant Materials, Experimental Design, and Locations

The most well-known maize seeds variety (Wanchuan-1306) was used in the current experiment, obtained from the Guangxi Wanchuan seed Industry Co. Ltd., China. The seeds were surface sterilized with 3% sodium hypochlorite (NaClO) for 30 min, and then washed 3–5 times in running tap water. After sterilization, seeds were kept at 25 °C in an incubator for 12 h. The healthy maize seeds were selected and five seeds were planted in each pot. The plastic pots (23.6 cm diameter, 24.5 cm height) were filled with 8 kg of soil mixture from arable topsoil added to each pot. The physical–chemical properties of the collected soil are shown in Table 1. The pots were arranged in randomized complete block design and placed in a glass shed under natural light conditions in the experimental station of Guangxi University, Nanning, China. At the maize crop’s early growth stage, the soil moisture content was retained at the normal field capacity (FC) of 75–80%. At the 5th leaf stage (V-5), progressive drought stress was imposed on the maize seedlings by withholding watering (40–45% FC), based on daily measurements of pot weight. Based on our previous research, an optimum level of MT (100 µM) was selected [25]. After 10 days of drought stress, 250 mL melatonin (100 µM) was applied as a root drenching to each pot for 3 days, and nitrogen was supplied at varying amounts in the form of urea at the time of sowing.

The experimental treatments designated for this study were (1) CK; Well-watered control without melatonin (MT), (2) DS; Drought stress without MT, (3) MT; Drought stress + 100 µM MT, (4) MT + N1; MT 100 µM + 200 kg N ha^−1^ (5) MT + N2; MT-100 µM + 250 kg N ha^–1^, and (6) MT + N3; MT-100 µM + 300 kg N ha^−1^. After 1 week of the MT treatment application, the different parameters were performed in each pot. The plant samplings for different tests were kept from each pot and then samples were immediately processed or stored at −80 °C for further analysis.

### 2.2. Sampling and Measurements

#### 2.2.1. Determination of Plant Biomass and Growth Attributes

From each treatment, the plant was sampled and divided into roots, shoots, and leaves to assess fresh- and dry biomass, whereas the stem diameter was measured with a vernier caliper. Root length and diameter were calculated by root image analysis using WinRHIZO 2003a software (Regent Instruments, Québec, QC, Canada). A tape meter was used to measure the plant height and leaf area per plant to detect growth changes. Fresh samples were weighed by an analytic balance to determine the fresh weight and then dried in an oven at 75 °C until constant weight was achieved and dry weight (DW) were determined. Root-to-shoot ratio was calculated using the following formula:

Root to shoot ratio = Root dry weight/Shoot dry weight(1)

#### 2.2.2. Determination of Chlorophyll Contents, Photosynthesis, and Chlorophyll Fluorescence

The chlorophyll content was measured using the method of Arnon [30]. One gram of fresh leaves was homogenized in 1 mL of 80% acetone, followed by centrifugation at 1500× *g* for 20 min at 4 °C. The absorbance of the supernatant was recorded at 645 nm and 663 nm for chlorophyll a and b, respectively, using a UV-spectrophotometer.

Photosynthetic gas exchange and chlorophyll fluorescence were measured by a portable infrared gas analyzer photosynthetic system LI-6800XT (LI-COR, Biosciences, Lincoln, NE, USA). After the 1 week of treatments, photosynthetic gas exchange parameters such as net photosynthetic rate, intercellular CO_2_, stomatal conductance, transpiration were measured between 10:00 and 12:00 pm on a sunny day. Whereas, the chlorophyll fluorescence Fv/Fm and ΦPSII were measured after dark acclimation for 30 min, the leaves were irradiated with 0.05 Hz high saturation light pulse for 600 s. A leaf chamber equipped with a red/blue LED light source was used. For the measurement of all treatments, the flow rate was constantly taken at 500 mL min^−1^ and CO_2_ concentration of ca. 400 µmol^−1^ under 1000 µmol m^−2^ S^−1^ photo-synthetically active radiation (PAR).

#### 2.2.3. Measurement of Antioxidant Enzymes Activities

For the determination of antioxidant enzymes activities, 0.2 g fresh leaf sample was obtained by removing the midrib portion. The sample was washed, dried, powdered, and homogenized in 5 mL chilled sodium phosphate buffer (50 mM, pH 7.8). The sample was centrifuged at 12,000× *g* for 20 min at 4 °C. The supernatant was used to measure superoxide dismutase, peroxidase, catalase, and ascorbate peroxidase enzymes activities, and results were expressed as U mg^−1^ min^−1^ FW.

Superoxide dismutase (SOD) activity was determined by measuring the photoreduction of nitroblue tetrazolium (NBT) at 560 nm using the method of Giannopolitis et al. [31]. About 20 μL of enzyme extract was added to the reaction mixture of 0.3 mL methionine (13 mM), 1.5 mL phosphate buffer (50 mM, pH 7.8), 0.3 mL EDTA-Na_2_ (0.1 mM), 0.3 mL NBT (750 mM), 0.3 mL riboflavin (20 M), and 0.3 mL distilled water. The reaction mixture tubes were put in 15 W lamps light for 10 min and then transferred to dark for 15 min, and absorbance was recorded at 560 nm using the UV spectrophotometer.

Peroxidase (POD) activity was determined at 25 °C guaiacol by the method of Ekmekci et al. [32]. In the presence of H_2_O_2_, POD catalyzes the conversion of guaiacol to tetra-guaiacol. Amounts of 0.1 mL H_2_O_2_ (300 mM), 2.7 mL potassium phosphate buffer (25 mM, pH 7.0), 0.1 mL guaiacol (1.5 percent *v*/*v*), 2 mM EDTA solution, and 0.1 mL enzyme extract made up the reaction mixture. A spectrophotometer was used to measure the absorbance at 470 nm every 30 s for up to 2 min.

The activity of the catalase (CAT) enzyme was determined using a previously described method that involves calculating the reduction in H_2_O_2_ absorption at 240 nm [33,34]. The reaction buffer contained 15 mM hydrogen peroxide (H_2_O_2_) and 50 mM potassium phosphate buffer at a pH of 7.0. Next, 100 µL of enzyme extract was added to the reaction mixture for the reaction initiation. The extinction coefficient of 40 mM^−1^ cm^−1^ was used to determine the quantity of H_2_O_2_ in the reaction mixture after 1 min, indicating the activity of CAT.

Ascorbate peroxidase (APX) activity was measured using the method of Ahmad et al. [35]. About 0.1 mM H_2_O_2_ was added to 3 mL of reaction solutions containing phosphate buffer (BPS; 50 mM, pH 7.0), 0.2 mM EDTA, 0.5 mM ascorbic acid, and 0.1 mL enzyme extract. The absorbance of the reaction mixture was read immediately at 290 nm with a UV- spectrophotometer.

#### 2.2.4. Determination of Nitrate Reductase (NR) and Glutamine Synthetase (GS) Activity

To measure and extract the NR activity, a nitrate reductase (NR) assay kit was used (BC0080, Beijing Solarbio Life Science & Technology Co., Ltd., Beijing, China). Fresh leaf (0.1 g) sample was extracted in 1 mL extraction solution and centrifuged for 10 min at 4000× *g* and supernatant was used to measure the NR activity at 340 nm using a UV-spectrophotometer.

A micro glutamine synthetase (GS) assay kit was used to extract and analyze glutamine synthetase (BC0915, Beijing Solarbio Life Science & Technology Co., Ltd., Beijing, China). 0.1 g fresh leaf sample that had been thoroughly crushed in liquid nitrogen was extracted with 1 mL extraction buffer. The mixture was centrifuged for 10 min at 8000× *g* at 4 °C. After centrifugation, the supernatant was used to measure GS activity and the absorbance was recorded at 540 nm with a UV-spectrophotometer.

#### 2.2.5. Determination of Reactive Oxygen Species (ROS) H_2_O_2_ and O_2_^−^

A previously described method by Zhang et al. [36] was used to determine H_2_O_2_ content. An amount of 0.1 g of leaf sample was pulverized and extracted with 5 mL of 0.1 percent TCA, then centrifuged for 15 min at 12,000× *g*. After that, 1 mL of 1 M potassium iodide and 0.5 mL of 10 mM phosphate buffer (pH 7.0) were added to 0.5 mL of supernatant. The absorbance was measured at 390 nm with a UV-spectrophotometer.

The superoxide (O_2_^−^) content was measured by the method of Zhang et al. [36]. An amount of 0.2 g of fresh leaf sample was extracted with 1 mL of BPS 65 mM, pH 7.8 and centrifuged for 10 min at 10,000× *g*. After that, 100 µL of supernatant was combined with 75 µL of BPS (pH 7.8) and 25 µL of 10 mM hydroxylamine hydrochloride and incubated at room temperature for 20 min. Subsequently, 1 mL of supernatant was mixed with 1 mL of 7 mM α-naphthalene diamine hydrochloride and 1 mL of 17 mM *a*-sulphanilamide in a reaction solution. After that, each tube was filled with 3 mL of ether and centrifuged at 5000× *g* at room temperature. With a UV spectrophotometer, the absorbance was measured at 540 nm and expressed as nmol min^−1^ g^−1^ FW.

#### 2.2.6. Statistical Analysis

Statistical analysis was performed using SPSS (SPSS10.0, Chicago, IL, USA). The standard deviation (±SD) of triplicates (*n* = 3, biological replicate) was calculated for each treatment. The least significance difference (LSD) tests were used to determine the significance of the differences between the mean values of each treatment at *p* ≤ 0.05. One-way analysis of variance was used to analyze the effects of the treatments on all data (ANOVA). In addition, Pearson’s correlation analysis was employed to determine the relationship between physiological and biochemical parameters. Graph Pad prism 7.00 was used to illustrate the figures.

## 3. Results

### 3.1. Effect of Melatonin and Nitrogen on Biomass Accumulation of Maize Seedlings

**Figure 1:**
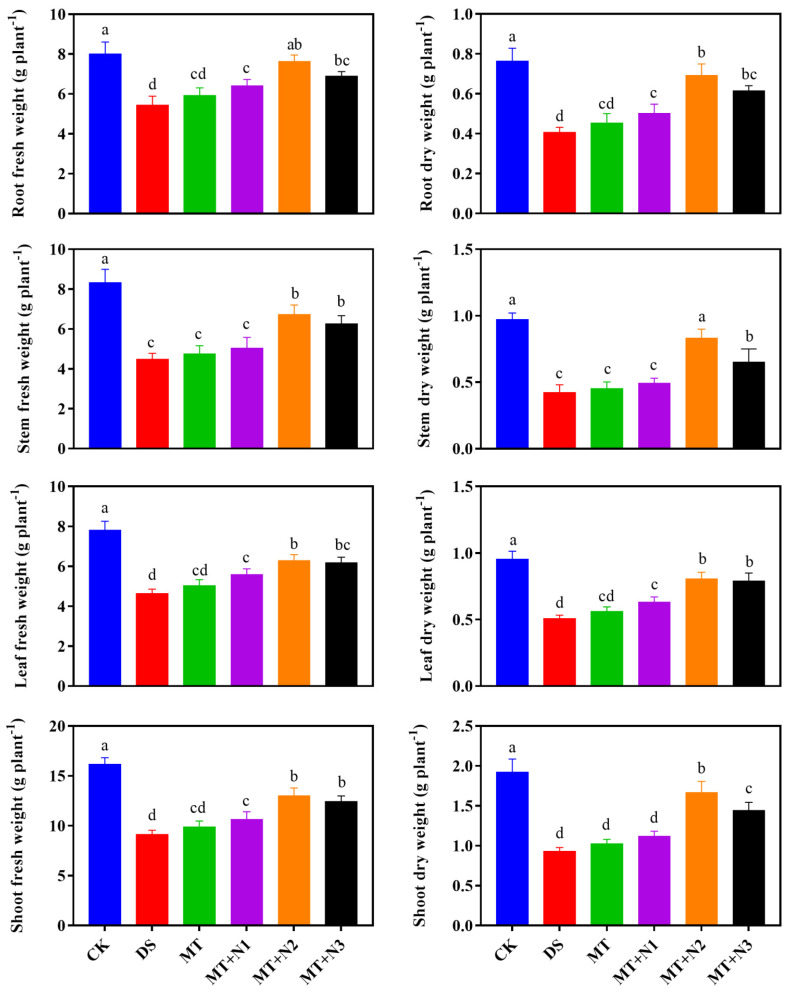
Fresh- and dry biomass accumulation of root, stem, leaf, and shoot of maize seedlings under drought-stress conditions in combination with melatonin in different levels of nitrogen. CK; Well-watered control without melatonin (MT), DS; Drought stress without MT, MT; Drought stress + 100 µM MT, MT + N1; MT 100 µM + 200 kg N ha^−1^, MT + N2; MT-100 µM + 250 kg N ha^−1^, and MT + N3; MT-100 µM + 300 kg N ha^−1^. Vertical bars represent ± S.D. (*n* = 3, biological replicates). Different letters indicate significant differences as determined by the LSD test (*p* ≤ 0.05).

The application of melatonin with nitrogen significantly affected the biomass accumulation of maize seedlings under drought-stress conditions compared with MT alone (Figure 1). Drought stress greatly reduced the fresh and dried root, stem, leaf, and shoot biomass of maize seedlings compared to CK (Figure 1). The detrimental effect of drought stress on biomass accumulation was considerably reduced by the combined treatment of melatonin and nitrogen. Our results showed that drought stress reduced the root fresh- and dry weight by 32.36% and 46.76% as compared to the CK. The applications of melatonin with nitrogen treatment (MT + N2) significantly enhanced the root fresh- and dry biomass by 40.53% and 70.11% compared to DS and MT. Similarly, the stem, leaf, and shoot fresh- and dry biomass of maize seedling were dramatically reduced by drought stress compared with CK, while melatonin with the application of nitrogen significantly improved these parameters. Our results, seen in Figure 1, demonstrate that treatment with MT + N2 significantly improved the stem fresh- and dry biomass by 50.52% and 84.30%, leaf fresh- and dry biomass by 35.14% and 59.88%, while the shoot fresh- and dry biomass by 42.68% and 79.19% followed by treatment with MT + N3 as compared to DS and MT. These results revealed that melatonin application combined with nitrogen significantly improved biomass accumulation under drought-stress conditions.

### 3.2. Effect of Melatonin and Nitrogen on Growth Attribute of Maize Seedlings

**Figure 2:**
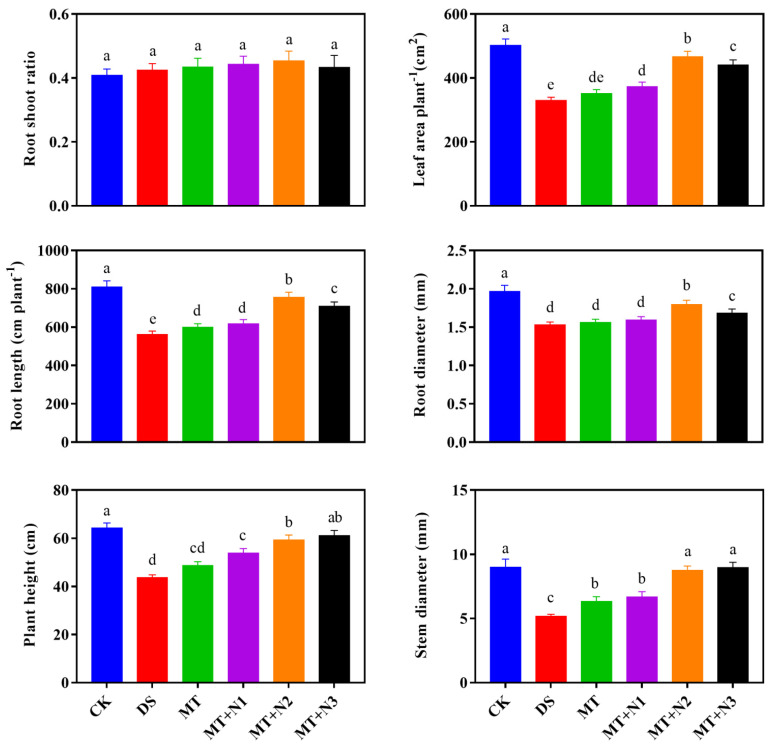
Root to shoot ratio, leaf area, root length and diameter, plant height, and stem diameter of maize seedlings under drought-stress conditions in combination with melatonin in different levels of nitrogen. CK; Well-watered control without melatonin (MT), DS; Drought stress without MT, MT; Drought stress + 100 µM MT, MT + N1; MT 100 µM + 200 kg N ha^−1^, MT + N2; MT-100 µM + 250 kg N ha^−1^, and MT + N3; MT-100 µM + 300 kg N ha^−1^. Vertical bars represent ± S.D. (*n* = 3, biological replicates). Different letters indicate significant differences as determined by the LSD test (*p* ≤ 0.05).

Exogenous melatonin with nitrogen treatment significantly affected the growth attributes of maize seedlings under drought-stress conditions compared with DS and MT. The root-to-shoot ratio was significantly similar in control and treated plants. Leaf area of maize seedling was evaluated in treated plants compared to drought stress. A larger leaf area per plant was observed in treatment MT + N2 by 41.54% compared with DS (Figure 2). Roots are the prime organ in plants that determines how much nutrients and water are absorbed. Drought stress drastically declined the root length and diameter by 30.46% and 22.15% of maize seedlings as compared to DS. Application of melatonin with nitrogen treatment MT + N2 significantly facilitated the root length and diameter by 34.25% and 17.18% as compared with DS (Figure 2). We also tested whether melatonin with the application of nitrogen had any effects on plant height and stem diameter of maize seedlings under drought stress. Maize seedlings exogenously treated with nitrogen appeared to be taller and had wider stems than the untreated plants under drought stress; however, statistically similar with the well-watered control. Our results exhibited that treatment MT + N2 had a taller plant height and greater stem diameter by 36.21% and 73.40% followed by M + N3 compared with DS (Figure 2).

### 3.3. Effect of Melatonin and Nitrogen on Chlorophyll and Fluorescence of Maize Seedlings

**Figure 3:**
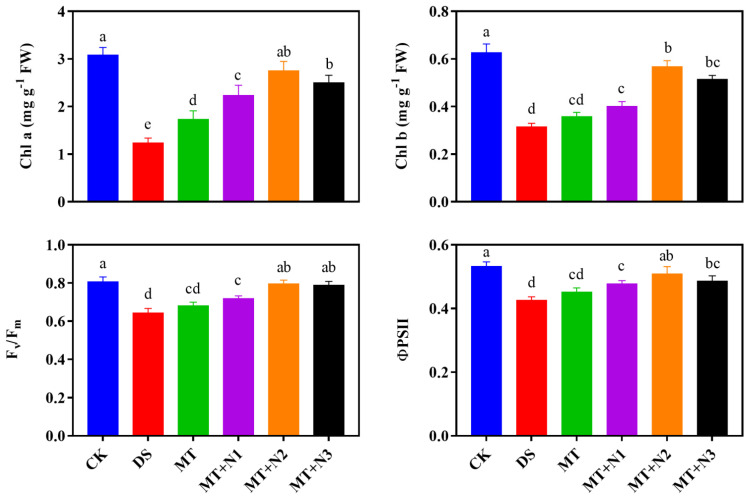
Chlorophyll a, b and chlorophyll fluorescence of maize seedlings under drought-stress conditions in combination with melatonin in different levels of nitrogen. CK; Well-watered control without melatonin (MT), DS; Drought stress without MT, MT; Drought stress + 100 µM MT, MT + N1; MT 100 µM + 200 kg N ha^−1^, MT + N2; MT-100 µM + 250 kg N ha^−1^, and MT + N3; MT-100 µM + 300 kg N ha^−1^. Vertical bars represent ± S.D. (*n* = 3, biological replicates). Different letters indicate significant differences as determined by the LSD test (*p* ≤ 0.05).

Concentrations of chlorophyll a and b, in maize seedlings were significantly decreased in the combined treatments of MT and N than CK. However, combined treatment of melatonin and nitrogen, regardless of its application method, significantly increased the chlorophyll pigments of maize seedlings under drought stress compared with MT alone. Our results showed that exogenous treatment of melatonin and nitrogen (MT + N2) significantly increased the chlorophyll a by 122.66% and chlorophyll b content by 80.63% followed by MT + N3 compared with DS (Figure 3).

Drought stress reduced the value of Fv/Fm and ΦPSII as compared to CK and treated plants. Our results showed that the combined application of melatonin and nitrogen increased the value of Fv/Fm and ΦPSII at a variable degree. The MT + N2 treatment showed a higher value of Fv/Fm and ΦPSII than DS and MT but was statistically similar to MT + N3. The value of Fv/Fm and ΦPSII were increased by 23.64% and 19.31% in treatment MT + N2 followed by MT + N3 as compared to DS (Figure 3).

### 3.4. Effect of Melatonin and Nitrogen on Photosynthetic Gas Exchange of Maize Seedlings

**Figure 4:**
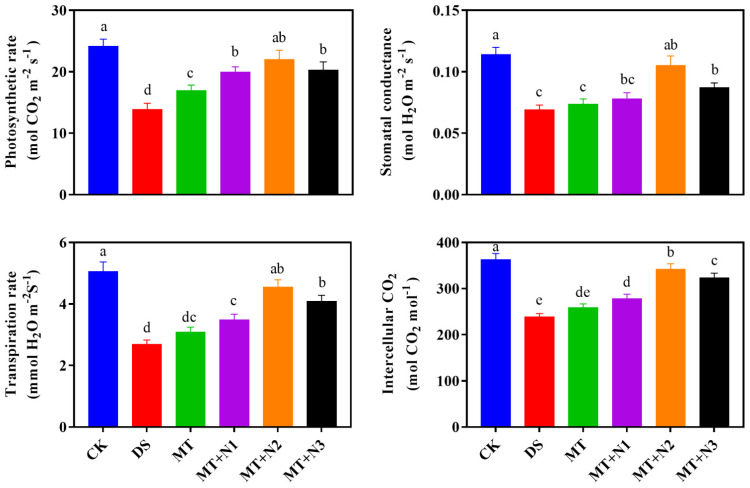
Photosynthetic gas exchange of maize seedlings under drought-stress conditions in combination with melatonin in different levels of nitrogen. CK; Well-watered control without melatonin (MT), DS; Drought stress without MT, MT; Drought stress + 100 µM MT, MT + N1; MT 100 µM + 200 kg N ha^−1^, MT + N2; MT-100 µM + 250 kg N ha^−1^, and MT + N3; MT-100 µM + 300 kg N ha^−1^. Vertical bars represent ± S.D. (*n* = 3, biological replicates). Different letters indicate significant differences as determined by the LSD test (*p* ≤ 0.05).

Drought stress caused a significant reduction in the photosynthetic gas exchange of maize seedlings. Exogenous treatment of melatonin and nitrogen alleviated more stress-induced damages in gas-exchange characteristics of maize seedlings than MT alone. MT + N1, MT + N2, and MT + N3 significantly improved the net photosynthetic rate by 44.03%, 58.32%, and 46.11% as compared with DS (Figure 4). However, melatonin with nitrogen treatment (MT + N2) significantly increased the stomatal conductance by 51.78%, transpiration rate by 69.31%, and intercellular CO_2_ by 43.51%, followed by MT + N3 compared with MT (Figure 4).

### 3.5. Effect of Melatonin and Nitrogen on Antioxidant Enzyme Activities of Maize Seedlings

**Figure 5:**
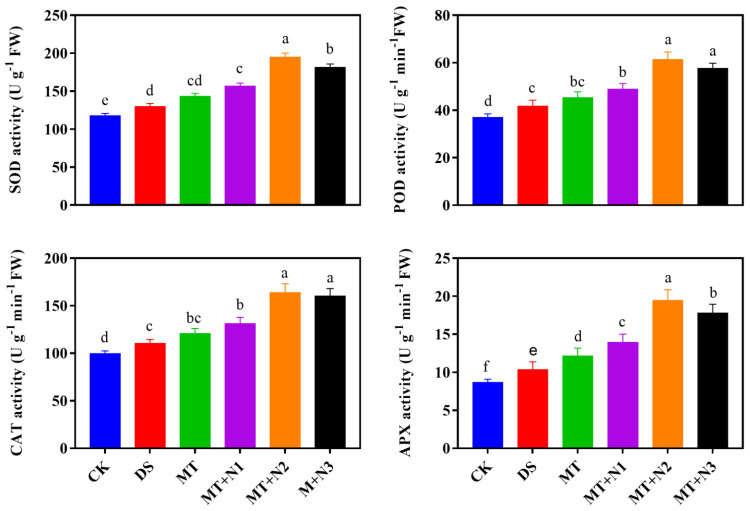
Antioxidant enzyme activities of maize seedlings under drought-stress conditions in combination with melatonin in different levels of nitrogen. CK; Well-watered control without (melatonin) MT, DS; Drought stress without MT, MT; Drought stress + 100 µM MT, MT + N1; MT 100 µM + 200 kg N ha^−1^, MT + N2; MT-100 µM + 250 kg N ha^−1^, and MT + N3; MT-100 µM + 300 kg N ha^−1^. Vertical bars represent ± S.D. (*n* = 3, biological replicates). Different letters indicate significant differences as determined by the LSD test (*p* ≤ 0.05).

Application of melatonin with nitrogen significantly improved antioxidant enzyme activity in drought-stressed maize seedlings as compared to untreated seedlings. Melatonin combined with various nitrogen levels improved the activity of different antioxidant enzymes in quite different ways than MT alone. SOD activity increased with increase in the concentration of nitrogen with melatonin. SOD activity increased by 50.03% in MT + N2 as compared to CK (Figure 5). The activity of POD and CAT showed the same increasing trend as those of SOD; however, MT + N2 and MT + N3 were statistically similar. The combined treatment of melatonin with nitrogen (MT + N2) improved the POD and CAT activity by 46.98% and 48.76% as compared to CK followed by MT + N3 (Figure 5). The activity of APX increased with increase in the treatment up to some extent, but the higher application of nitrogen with melatonin reduced the APX activity. Under drought-stress conditions, higher APX activity was recorded in treatment MT + N2 by 88.19% as compared to CK (Figure 5).

### 3.6. Effect of Melatonin and Nitrogen on NR and GS Activity of Maize Seedlings

**Figure 6:**
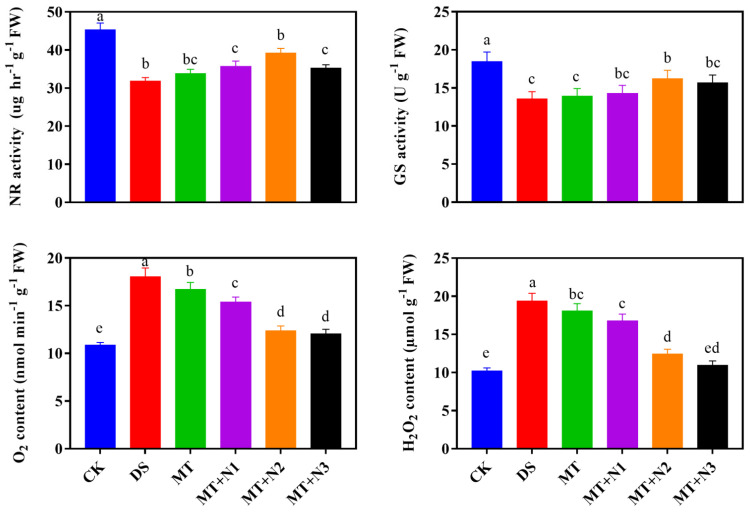
Nitrogen metabolism enzyme glutamine synthetase (GS), nitrate reductase (NR) activities, and reactive oxygen species (ROS) of maize seedlings under drought-stress conditions in combination with melatonin in different levels of nitrogen. CK; Well-watered control without melatonin (MT), DS; Drought stress without MT, MT; Drought stress + 100 µM MT, MT + N1; MT 100 µM + 200 kg N ha^−1^, MT + N2; MT-100 µM + 250 kg N ha^−1^, and MT + N3; MT-100 µM + 300 kg N ha^−1^. Vertical bars represent ± S.D. (*n* = 3, biological replicates). Different letters indicate significant differences as determined by the LSD test (*p* ≤ 0.05).

Nitrogen metabolism enzymes NR and GS activity in maize seedling leaves were significantly affected by the combined application of melatonin and nitrogen under drought-stress conditions. Melatonin with nitrogen treatment (MT + N2) increased the NR activity by 6.74%, and GS activity by 19.67% as compared to DS and MT (Figure 6). Overall results showed that melatonin application under drought stress improves the antioxidant enzyme and increases the nitrogen metabolism activity.

### 3.7. Effect of Melatonin on Reactive Oxygen Species (H_2_O_2_ and O_2_^−^) of Maize Seedling

The H_2_O_2_ and O_2_^−^ are produced prominently in plants and as an indicator of ROS scavenges of plants under drought-stress conditions. Our finding indicated that drought stress dramatically increased the H_2_O_2_ and O_2_^−^ concentration under drought-stress conditions as compared to CK and treated plants. Regardless of the melatonin with the nitrogen application process, the generation of H_2_O_2_ and O_2_^−^ steadily decreased with the increase in its concentration under drought-stress conditions. Our results demonstrated that the application of melatonin and nitrogen treatments MT + N1, MT + N2, and MT + N3 reduced the H_2_O_2_ by 13.32%, 35.96%, and 43.64%, and the O_2_^−^ accumulation by 14.83%, 31.47%, and 33.31% as compared to DS (Figure 6).

### 3.8. Correlation Analysis

**Figure 7:**
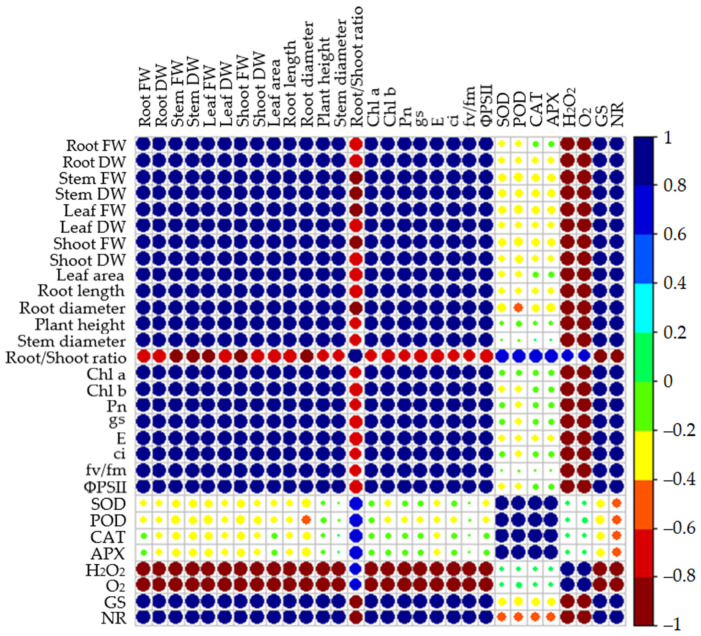
Correlation analyses of the inspected parameters of maize seedlings under drought-stress conditions in combination with melatonin in different levels of nitrogen. Root fresh weight (Root FW), root dry weight (Root DW), stem fresh weight (Stem FW), stem dry weight (Stem DW), leaf fresh weight (Leaf FW), leaf dry weight (Leaf DW), leaf area, root length, root diameter, plant height, stem diameter, root-to-shoot ratio, chlorophyll a,b (Chl a,b), net photosynthetic rate (gs), intercellular CO_2_ (Ci), stomatal conductance (gs), and transpiration rate (E), maximal quantum efficiency of PSII photochemistry (Fv/Fm), the effective quantum yield of PSII photochemistry (ΦPSII), superoxide dismutase (SOD), peroxidase (POD), catalase (CAT), ascorbate peroxidase (APX), hydrogen peroxide (H_2_O_2_) superoxide (O_2_), glutamine synthetase (GS), and nitrate reducates (NR) activities. Different colours on the scale from green to dark blue indicate positive correlation while light green to dark red indicate negative correlation.

The correlation was carried out to find out the relationship between physiological and biochemical parameters of maize seedlings under drought-stress conditions. Pearson’s correlation analysis results revealed that fresh- and dry biomass, root length and diameter, plant height, leaf area, chlorophyll fluorescence, and photosynthetic gas exchange parameter were significantly high and positively correlated with each other, while the root-to-shoot ratio showed negative correlation (Figure 7). Moreover, antioxidant enzyme activity had a negative correlation, while the nitrogen metabolisms GS and NR had a positive correlation. In addition, the ROS had a strongly negative correlation with the growth parameter of maize seedlings under drought-stress conditions (Figure 7).

## 4. Discussion

Drought stress is one of the major limitations which leads to various physiological and biochemical responses in plants. Drought stress causes abnormal plant growth and development by inhibiting cell proliferation and expansion [2,37]. In the present study, the growth of maize seedlings was markedly inhibited after the plants were exposed to drought stress. Application of melatonin as a soil drenching reduced the severity of drought-induced growth inhibition and enhanced maize drought tolerance in terms of promoting the plant’s growth under drought stress [38]. Melatonin is an evolutionarily highly conserved chemical and has a wide range of functions in plants. Previous research revealed that melatonin and nitrogen application improved plant growth and development under abiotic stress conditions [8,28,35]. However, nitrogen impact depends on the severity of drought stress; nitrogen levels simply reduce the inhibitory effect of drought stress on the growth and development of plants [8,39]. Inhibited growth is a key sign of plants that have been exposed to environmental stress [40,41]. Our study observed that the value of fresh- and dry biomass accumulation of root stem, leaf, and the shoot was significantly lower under drought-stress conditions. The melatonin combined with the optimum nitrogen level (MT + N2) treated plants greatly improved the fresh- and dry biomass and growth attributes under drought-stress conditions. Numerous researchers also reported that the application of melatonin and nitrogen increased the biomass accumulation and growth characteristics and endorsed the improved carbon assimilation due to enhanced photosynthetic capacity [40,42]. As a result of our research, melatonin combined with nitrogen significantly improves seedling adaptation to drought stress by alleviating the suppression of growth characteristics produced by drought stress. Moreover, the optimum level of melatonin with nitrogen (MT + N2) treatment resulted in denser roots with greater root length, root diameter, taller plant height, and stem diameter as compared with drought stress (Figure 1). Our results agree with the previous studies reporting that melatonin enhanced plant biomass and root length of tobacco and maize seedlings under abiotic stress conditions [43,44]. The presence of endogenous promoters was likely increased by melatonin and nitrogen treatment resulting from the deeper roots. Auxin-modulated physiological systems control melatonin-induced root development, regulating water absorption and triggering irreversible cell wall extension [38]. The source of the value-added root increase appears to be the melatonin-mediated higher adaptability of treated plants for more effective uptake of soil water and nutrients that are transported to the aerial parts of plants [42].

Plant growth and development were inhibited by water scarcity, which reduced the photosynthetic efficiency and biomass while increasing the accumulation of ROS levels. However, the application of melatonin increased the growth and photosynthetic pigments and antioxidant enzyme activity by reducing the ROS under drought-stress conditions [45]. Chlorophyll is the key leaf pigments involved in photosynthesis, a principal physicochemical process that governs the synthesis of organic compounds using light energy, which is the base of plant growth and development. Drought stress reduces leaf area, affects photosynthetic pigments, reduces photosynthesis, and ultimately inhibits plant growth [18,46]. Our results demonstrated that the application of melatonin and nitrogen reduced the negative effects of drought stress, increased the leaf area per plant, and improved the amount of chlorophyll content as compared to the drought-stressed plants. These findings show that melatonin application improved biosynthesis and reduced the degradation of chlorophyll pigments, which was impeded by drought stress. Chlorophyll fluorescence has become a valuable approach for researching plant photosynthetic capabilities under abiotic stress conditions. According to certain research, severe or long-term dryness causes photo-inhibition in the PSII reaction center [41,46,47]. Our results are in agreement with these observations: drought stress drastically reduced the Fv/Fm and ΦPSII as compared to the well-watered control. However, melatonin combined with an optimum level of nitrogen treatment MT + N2 significantly improved the Fv/Fm and ΦPSII value as compared with DS and MT. Drought stress caused severe damage to the photosynthetic apparatus in maize seedlings, as evidenced by the decrease in Fv/Fm and ΦPSII. Furthermore, melatonin has enhanced photosynthetic efficiency in higher plants under abiotic stress conditions [13,48]. Drought stress leads plants to close their stomata to conserve water, which results in large reductions in stomatal conductance and, as a result, stomatal limitation of photosynthesis [25]. Previous research has found that an optimum amount of melatonin improved stomatal functioning by allowing plants to reopen their stomata under abiotic stress conditions [11,28]. Our results showed that photosynthetic gas exchange parameters were dramatically reduced under drought-stress conditions due to stomatal limitations. However, melatonin combined with nitrogen treatment (MT + N2) improved the gas exchange parameter as compared with DS and MT. As a result, melatonin alleviation of stomatal restriction contributed to increased photosynthetic gas exchange under drought stress [37]. This finding suggested that an adequate level of nitrogen with melatonin application boosts photosynthetic efficiency and productivity in the leaves of maize seedlings under drought-stress conditions.

Antioxidant enzyme activity regulation is an innate plant response to prevent oxidative stress generated by various external biotic and abiotic stress factors [49,50]. Plants have evolved an effective defense mechanism of antioxidants enzymes activities such as SOD, POD, CAT, and APX to deal with oxidative damages. Superoxide dismutase is a key enzyme that controls O_2_^−^ levels in leaves and is involved in the initial stage of the cellular defense mechanism, while CAT and APX activity regulates the H_2_O_2_ accumulation and reduce it to H_2_O under abiotic stress conditions [18,19]. In the present study, our results demonstrated that the treatment MT + N2 significantly improves the antioxidant enzyme activities compared with control. Remarkably, the harmful effects of drought stress were reduced by the application of melatonin with nitrogen, which considerably increased the antioxidant enzymes activities. The various antioxidant enzymes indicated a different increasing pattern by increasing the treatment application at an optimum level applied as a drenching to roots under drought-stress conditions. Previous research indicated that combined treatment of melatonin with nitrogen improved stress tolerance in many crops by protecting the photosynthetic machinery, enhancing antioxidant capacity, and improving water-holding capacity [8,51]. We also found that exogenous treatment of melatonin combined with nitrogen enhanced the antioxidant enzyme activities and improved the nitrogen metabolism enzymes under drought-stress conditions. In general, the nitrate reductase (NR) and glutathione synthase (GS) enzymes are involved in N metabolism [52,53]. NR is a substrate-inducing enzyme primarily activated at the transcriptional level and triggered by NO_3_^−^-N, carbohydrates, and light, among other things [54]. Furthermore, GS plays a key function in plant nitrogen metabolism. In the current study, the melatonin and N treatments significantly impacted the GS and NR activity in the leaf under drought stress. Our results revealed that melatonin combined with nitrogen had a positive effect on the NR and GS enzymatic activity under drought-stress conditions. Our findings are comparable with previous reports, which found that melatonin increased the activities of many enzymes involved in N metabolism, including NR and GS activity in maize plants under abiotic stress conditions [13,28]. Furthermore, Liang et al. [55] also exhibited that long-term melatonin treatment at 100 M increased enzymes involved in N metabolism in apple plants. Likewise, the previous study perceived that melatonin treatment also improves the NR and GS activity in wheat crops compared to treatment under abiotic stress conditions [13].

The plant’s defense system is easily eliminated under harsh environmental conditions, such as drought. A considerable amount of ROS accumulates under drought-stress conditions, but melatonin combined with nitrogen significantly reduces the ROS levels [2,8,55]. Our results showed that under drought-stress conditions, H_2_O_2_ concentration and O_2_^−^ production rates were much higher in maize leaves, whereas nitrogen combined with melatonin supplementation significantly reduced ROS accumulation. When the degree of drought stress is moderate, an appropriate application of nitrogen and melatonin enhances the activities of antioxidant enzymes. These enzymes prevent lipid peroxidation and protect the integrity of the cell membrane by reducing the accumulation of reactive oxygen species. Melatonin is a broad-spectrum antioxidant and free radical scavenger that may eliminate ROS directly when produced under stress conditions [24,46]. Therefore, based on the present study’s finding, the positive effect of melatonin combined with an optimum nitrogen level (MT + N2) improved growth, increased the chlorophyll pigments, activated antioxidant mechanisms, nitrogen metabolism enzyme, and reduced ROS accumulation maize seedling under drought-stress conditions.

## 5. Conclusions

Drought stress hinders plant growth attributes and their physiological processes. Exogenous application of melatonin is an effective strategy to mitigate the deleterious effects of drought stress. Our results indicated that melatonin application with optimum nitrogen level comprehensively alleviated drought stress of maize seedlings by improving its various morphological and physicochemical attributes. Therefore, the drenching method of melatonin with an appropriate nitrogen level could effectively be used to alleviate drought stress and improve the seedling growth of maize. Overall results indicated that melatonin with nitrogen (MT + N2) improved the growth of maize seedlings under drought-stress conditions.

## Figures and Tables

**Table 1:** Physical and chemical composition of the soil of the experimental pots.

Parameters	Values
Soil organic matter	14.61 g/kg
Available nitrogen	0.88 g/kg
Available phosphorus	48.85 g/kg
Available potassium	96.37 mg/kg
Soil pH	6.83
Water holding capacity	30.31%

## Data Availability

The data presented in this study are available in the graphs and table provided in the manuscript.

## Abbreviations

FC	Field capacity
N	Nitrogen
GS	Glutamine synthetase
NR	Nitrate reducates
CK	Well-watered control;
DS	Drought stress
MT	Melatonin
M + N	Melatonin plus Nitrogen
DW	Dry weight
ROS	Reactive oxygen species
SOD	Superoxide dismutase
POD	Peroxidase
CAT	Catalase
APX	Ascorbate peroxidase
AsA-GSH	Ascorbate glutathione
GSH	Glutathione
ASA	Ascorbate
O_2_	Superoxide
H_2_O_2_	Hydrogen peroxide
